# Pan-genomic characterization of high-risk pediatric papillary thyroid carcinoma

**DOI:** 10.1530/ERC-20-0464

**Published:** 2021-04-06

**Authors:** Adam Stenman, Samuel Backman, Klara Johansson, Johan O Paulsson, Peter Stålberg, Jan Zedenius, C Christofer Juhlin

**Affiliations:** 1 Department of Oncology-Pathology, Karolinska Institutet, Stockholm, Sweden; 2 Department of Molecular Medicine and Surgery, Karolinska Institutet, Stockholm, Sweden; 3 Department of Breast, Endocrine Tumors and Sarcoma, Karolinska University Hospital, Stockholm, Sweden; 4 Department of Surgical Sciences, Uppsala University, Uppsala, Sweden; 5 Department of Pathology and Cytology, Karolinska University Hospital, Stockholm, Sweden

**Keywords:** whole-genome sequencing, RNA sequencing, mutation, pediatric thyroid cancer, papillary thyroid carcinoma

## Abstract

Pediatric papillary thyroid carcinomas (pPTCs) are often indolent tumors with excellent long-term outcome, although subsets of cases are clinically troublesome and recur. Although it is generally thought to exhibit similar molecular aberrancies as their counterpart tumors in adults, the pan-genomic landscape of clinically aggressive pPTCs has not been previously described. In this study, five pairs of primary and synchronously metastatic pPTC from patients with high-risk phenotypes were characterized using parallel whole-genome and -transcriptome sequencing. Primary tumors and their metastatic components displayed an exceedingly low number of coding somatic mutations and gross chromosomal alterations overall, with surprisingly few shared mutational events. Two cases exhibited one established gene fusion event each (*SQSTM1*-*NTRK3* and *NCOA4*-*RET*) in both primary and metastatic tissues, and one case each was positive for a *BRAF* V600E mutation and a germline truncating *CHEK2* mutation, respectively. One single case was without apparent driver events and was considered as a genetic orphan. Non-coding mutations in cancer-associated regions were generally not present. By expressional analyses, fusion-driven primary and metastatic pPTC clustered separately from the mutation-driven cases and the sole genetic orphan. We conclude that pPTCs are genetically indolent tumors with exceedingly stable genomes. Several mutations found exclusively in the metastatic samples which may represent novel genetic events that drive the metastatic behavior, and the differences in mutational compositions suggest early clonal divergence between primary tumors and metastases. Moreover, an overrepresentation of mutational and expressional dysregulation of immune regulatory pathways was noted among fusion-positive pPTC metastases, suggesting that these tumors might facilitate spread through immune evasive mechanisms.

## Introduction

The incidence of pediatric thyroid cancer is estimated to be 4–5 cases per 100,000 children, making thyroid cancer the most common pediatric endocrine malignancy (Howlader *et al.* 2020). The bulk of cases are follicular-cell derived neoplasia denoted as pediatric papillary thyroid carcinoma (pPTC) or pediatric follicular thyroid carcinoma, while small subsets of cases are derived from the parafollicular C-cells; namely pediatric medullary thyroid carcinoma. As in adult patients, the prognosis is usually excellent from a mortality perspective but the morbidity can be troublesome – with numerous recurrences and visits to the operation theater for some of these children (Christison-Lagay *et al.* 2020, de Jong *et al.* 2020, Lebbink *et al.* 2020, Sugino *et al.* 2020*a*,*b*). To pinpoint which cases that need vigilant follow-up and intensified treatment upfront is not always possible, in part due to the general lack of scientific literature regarding pediatric thyroid cancer. As of this, specific considerations are needed when tackling pediatric thyroid cancer in the clinical setting, not least manifested through the advent of detailed American Thyroid Association (ATA) guidelines for treating children with thyroid nodules (Francis *et al.* 2015). From a genetic standpoint, gene fusion events are significantly overrepresented in pediatric thyroid cancer as opposed to adult tumors, whereas the latter entity exhibits more frequent mutational events compared to tumors in children (Picarsic *et al.* 2016, Mostoufi-Moab *et al.* 2018, Paulson *et al.* 2019, Pekova *et al.* 2020, Potter *et al.* 2020). For PTCs specifically, the most common driver event is the *BRAF* V600E mutation, which is present in around 50–60% of all PTC cases in adults, while only present in 25–30% of pPTCs (Paulson *et al.* 2019). Although this mutation has been designated as a predictor of worse outcome in adult cases, the prognostic role of the V600E mutation in pPTC is somewhat more debatable (Henke *et al.* 2014). Instead, pPTCs are generally driven by gross chromosomal events. Around 2000 pPTCs with associated molecular data have been published to date, and *RET* gene fusions to various gene partners (most commonly *CCDC6* and *NCOA4*, termed *PTC1* and *PTC3* respectively) seem to be the most common fusion event in this tumor category, detected in approximately 25–30% of all cases – thereby alone occurring at similar frequencies as the *BRAF* V600E mutation (Paulson *et al.* 2019). Interestingly, an association between *RET* gene fusions and radiation exposure is noted, but can hardly explain the increased incidence of pPTC in the Western hemisphere as of 2020 (Cordioli *et al.* 2015). Other fusion types include *NTRK1* and *2*, *BRAF* and *ALK*, which are less common (occurring in 10, 10 and 5% of cases, respectively) (Picarsic *et al.* 2016, Paulson *et al.* 2019, Potter *et al.* 2020). Other genetic events associated to PTCs in the adult population, such as RAS gene family mutations, are much rarer in pPTCs (<5% of cases) (Paulson *et al.* 2019).

Despite recent advances in the field of molecular genetics, to our knowledge, there has been no previous pan-genomic characterization focusing solely on pediatric thyroid cancer specimen using whole-genome sequencing (WGS) and RNA sequencing (RNAseq) analyses of coupled normal-tumor-metastases. As WGS widely surpasses conventional whole-exome sequencing (WES) in terms of interrogated bases, we saw an opportunity to identify genetic mechanisms outside the grasp of conventional sequencing of coding regions by (1) identification of potentially important genetic aberrations in non-coding regions, (2) high-resolution coverage of the copy number alteration and fusion gene landscapes and (3) give new insight into tumor phylogenetic changes in primary tumors vs metastases. We have, therefore, successfully interrogated DNA and RNA from constitutional, primary tumor and metastatic tissues from five pPTC patients presenting with a rather large tumor burden in terms of lateral lymph node metastases, of which some also exhibited recurrent disease, either locally or distant. As of this, these cases represent high-morbidity cases at our institution.

## Materials and methods

### Patient cohort and associated clinical characteristics

The Karolinska University Hospital in Solna, Sweden is the largest tertiary referral center for thyroid cancer in the Nordic region with an approximate catchment area of 2 million inhabitants. By preliminary screenings of the pathology database at our department, we have so far diagnosed 1552 primary PTCs through histopathology from 1992 to the current date. Of these, 28 were pPTC cases (18 years old or less) – representing 1.8% of the total amount of cases. From this cohort, we selected a total of five patients with pPTC based on their availability regarding fresh frozen constitutional, primary tumor and metastatic tumor tissues in our biobank repositories, the pTNM status (only allowing at least a pT3 stage and an N1b stage respectively), a high metastatic burden (loosely defined by the authors as a positive lymph node ratio of >0.4 in central and lateral cervical compartments combined) as well as giving priority to any case with recurrent disease and distant metastases. In the most recent ATA guidelines for the pediatric population, the ATA pediatric high-risk level is defined by regionally extensive disease (extensive N1b), which all cases included in this study exhibit (Francis *et al.* 2015).

The clinical characteristics of these five cases are detailed in Table 1. Specifically, two cases were conventional PTCs, two cases were diffuse sclerosing variant PTCs and one single case was a follicular variant PTC (Supplementary Figs 1, 2, 3, 4 and 5, see section on supplementary materials given at the end of this article). All tumors were diagnosed using histopathology and the World Health Organization (WHO) criteria used at the time of diagnosis and re-evaluated using the updated criteria dictated in the most recent volume (Lloyd *et al.* 2017). All samples were obtained with informed patient consent and with approval from the Swedish Ethical Review Board (approval no. EPN 2015: 959-31).

**Table 1:** Clinical characteristics of the five children with papillary thyroid carcinoma included in the study.

Case ID	1	2	3	4	5
Age at surgery	9	11	13	14	15
Sex	F	M	F	F	M
Primary tumor size (mm)	48	30	75	42	30
PTC subtype	DSV-PTC	FV-PTC	DSV-PTC	Conventional	Conventional
Ki-67 proliferation index	6%	<1%	3%	6%	1%
ETE	No	Yes	Yes	Yes	Yes
Multifocality	No	No	Yes	No	Yes
Central and lateral lymph node status	22/45	3/3	35/83	17/29	12/22
pTNM*	pT3N1b	pT3N1b	pT3(m)N1b	pT3N1b	pT3(m)N1b
Recurrences	Local met	None	None	Local met	Distant met
Alive	AWOD	AWOD	AWOD	AWOD	AWD

*As according to AJCC version 7.

AWD, alive with disease; AWOD, alive without disease; DSV, diffuse sclerosing variant; ETE, extrathyroidal extension; FV, follicular variant; Met, metastasis; PTC, papillary thyroid carcinoma.

### DNA/RNA extraction and tumor tissue content testing

Fresh frozen tumor tissue from primary tumor and synchronous lymph node metastases were retrieved from our biobank repositories, and extraction of DNA and RNA was performed using the DNeasy Blood & Tissue Kit and RNeasy Plus Mini Kit respectively, following the protocol of the manufacturer (QIAGEN). In addition, a small piece of tissue was also cut for fixation in formalin, followed by paraffin embedment and sectioning for tumor tissue content analysis using conventional light microscopy. The tumor cell percentages were on average 60%, ranging from 40 to 80%. For constitutional tissues, leukocyte DNA was ordered from the blood biobank at Karolinska Institutet for four out of five patients. These samples were preoperatively drawn from each patient and saved in a −86°C freezer. For a single case (patient 5), no peripheral blood was available in the biobank and we instead extracted DNA from fresh frozen normal thyroid tissue acquired in conjunction to grossing of the primary tumor. This normal tissue sample was also investigated regarding representation testing using light microscopy, to verify the presence of normal thyroid tissue (80% normal thyroid tissue, 20% stroma) without tumor cell contamination.

### Whole-genome sequencing and associated bioinformatics

Following library preparation with 1 µg of DNA using the Illumina TruSeq PCR-free and a target insert size of 350 base pairs, samples were sequenced using the NovaSeq 6000 S4 platform. The generated data were analyzed using the Sarek pipeline (Garcia *et al.* 2020). Briefly, the generated reads were mapped to the GRCh38 human reference genome. Base Quality Score Recalibration and Deduplication were performed followed by somatic variant calling using Mutect2 for SNVs and indels, and Manta for structural variants (Chen *et al.* 2016). Copy number analysis was performed using ASCAT (Van Loo *et al.* 2010).

Somatic variants passing all MuTect2 filters and predicted to cause a frameshift, inframe insertion/deletion, amino acid substitution, stop gain, stop loss, start loss, or affecting a canonical splice acceptor or donor were included in the coding somatic mutation call set.

For non-coding variants, a similar strategy to Sakthikumar * et al.* was employed (Sakthikumar *et al.* 2020). First, false-positive somatic mutations were likely excluded by filtering out variants with allele frequencies of 0.1% or more in gnomAD (Karczewski *et al.* 2020). Genome-wide GERP scores for GRCh38 calculated against 103 mammals were obtained from Ensembl. A custom Python script using the pyBigWig library was used to extract variants with a GERP-score of at least 2. We further extracted variants overlapping a putative regulatory region from the 20190329 Ensembl regulatory build. These variants affecting conserved bases in putative/confirmed regulatory regions were used for downstream analyses. Additionally, the *TERT* and *PLEKHS1* promoter regions were specifically interrogated by extracting all somatic variants in the regions chr5:1294788-1296488 and chr10:113748262-113754262. Germline variants were called using HaploTypeCaller and annotated with gnomAD allele frequencies using vcfanno (Pedersen *et al.* 2016).

### RNA sequencing

Library preparation was performed using the Illumina TruSeq strand-specific RNA libraries (using poly-A selection) from a total of ten RNA samples (five primary pPTCs, five metastatic pPTCs). Samples were sequenced in multiplex on the Illumina NovaSeq S4, PE 2 × 150 bp. Data processing was performed via demultiplexing and gold standard quality control. Quantification was performed using kallisto (Bray *et al.* 2016), and differential expression analyses using sleuth (Pimentel *et al.* 2017). Differential analyses were performed at the gene level, using the Wald test and genes with a q-value <0.05 were considered differentially expressed. For clustering based on gene expression, the 3000 genes with the highest dispersion index (variance divided by mean) across the cohort were extracted. Hierarchical clustering and heatmap plotting were performed using the Seaborn ClusterMap function after calculation of gene-wise z-scores. For gene fusion analyses, kallisto was run with the fusion option, and the output was further processed using pizzly (https://www.biorxiv.org/content/10.1101/166322v1.article-info). Putative gene fusions were compared with the structural variants called by Manta, and only fusions with some evidence on both the DNA and the RNA level were retained in the final call set.

### Analysis of the cancer genome atlas (TCGA) dataset

To evaluate the mutational status, copy number alterations and mRNA expressional results of the coding somatic variants of cases 1 and 3 (Supplementary Table 1) in an extended cohort, data from 414 PTC patients from the cBioPortal for cancer genomics were analyzed (Cerami *et al.* 2012, Gao *et al.* 2013). Complete samples available for all analyses *n* = 388.

## Results

### Whole-genome sequencing quality parameters

Fifteen samples were successfully sequenced using WGS, including five primary pPTCs, five corresponding lymph node metastases and five constitutional tissues (blood *n*  = 4, normal thyroid tissue *n*  = 1). The total million reads per tumor sample (*n* = 10) were 802.8 on average, ranging from 670.44 to 998.64. The aggregated percentage of bases that had a quality score more than the Q30 value was 93.1% on average, ranging from 92.8 to 93.49%. The Q30 mark is equal to an inferred base call accuracy of 99.9%. For the constitutional tissues, the total million reads per tumor sample were 817.6, with on average 93.4% of bases surpassing the Q30 value.

### Somatic mutational overview

**Figure 1:**
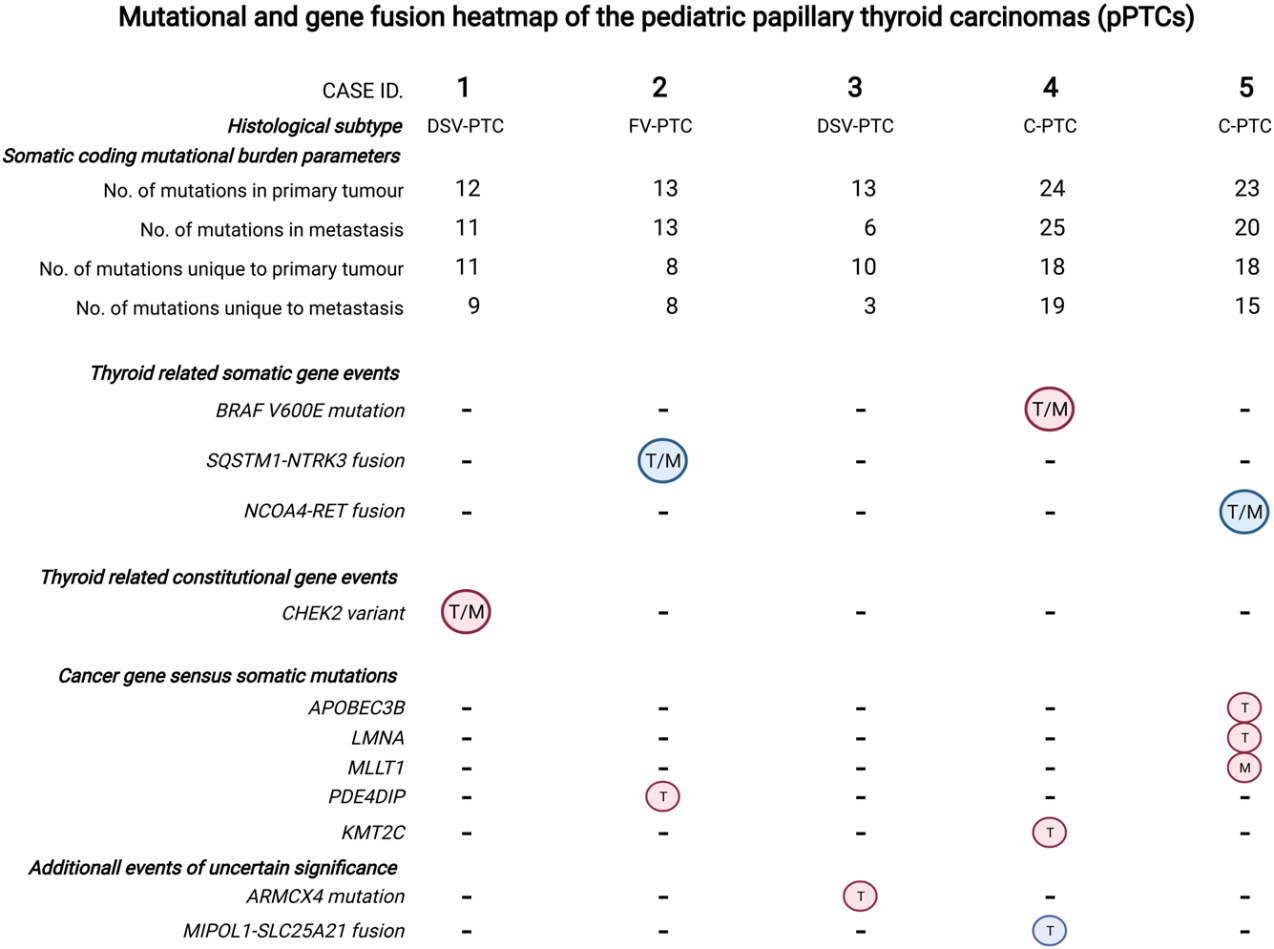
Mutational and gene fusion heatmap of the pediatric papillary thyroid carcinomas (pPTCs). Coding mutation burden (somatic protein-altering and splice-site single nucleotide variants) and key genetic findings using whole-genome sequencing are shown. In the heatmap, red circles represent mutational events and blue circles denote gene fusions. DSV-PTC, diffuse sclerosing variant PTC; FV-PTC, follicular variant PTC; C-PTC, conventional PTC; T, occurring in the primary tumor; M, occurring in the lymph node metastasis; T/M, occurring in both primary tumor and lymph node metastasis. Image created with BioRender.com.

After the filtering of constitutional variants through the constitutional sequencing data and removal of less impactful variants using the MuTect2 filter, a total of 85 somatic protein-altering and splice-site single nucleotide variants (SNVs) were found among the primary pPTCs (on average 17 variants per case, range 12–24). For the corresponding metastatic samples, the total number of coding events was 75, on average 15 SNVs per case. The mutational burden and main events are illustrated in Fig. 1, and the complete list of somatic SNVs in both primary and metastatic pPTCs is available in Supplementary Table 1.

When looking specifically for alterations in thyroid-related genes (any reported mutation from the previous pan-exomic Cancer Genome Atlas study of 500 PTC cases), case 4 was found to harbor a *BRAF* V600E mutation in both primary and metastatic samples (Cancer Genome Atlas Research Network 2014). In all other samples, no somatic mutations previously reported in the TCGA paper (which is largely based on adult PTC cases) were found. When, instead filtering the total SNV list to the Catalogue of Somatic Mutations in Cancer (COSMIC) Cancer Gene Census (CGC) database of genes reported mutated in cancer, we found a few additional genes with missense mutations; *APOBEC3B* (two different mutations; p.Arg396His and p.Val399Gly) and *LMNA* (p.Thr333Pro) in the primary tumor of case 5, *MLLT1* (p.Lys269Gln) in the corresponding metastatic sample. Moreover, *PDE4DIP* (p.Gln147Glu) was found mutated in the primary tumor of case 2, and a mutation in the histone modifier *KMT2C* (p.Gly845Glu) was observed in the primary tumor of case 4 (Fig. 1). However, no recurrent gene mutations or mutations in recurrent CGC genes were observed. Finally, when manually scrutinizing mutated genes and comparing to findings in the COSMIC database, case 3 (with no credible thyroid-related driver event) was found to exhibit a somatic *ARMCX4* mutation (c.5474C>T, p.Ala1825Val) in the primary tumor (Fig. 1). Although not a tier 1 CGC gene, *ARMCX4* mutations have been reported in 43 thyroid cancer specimens out of >1000 sequenced cases (COSMIC study COSU676). Our specific variant was not found among the reported mutations, although several recurrent mutations in neighboring amino acid positions were observed (p.A1821V, p.E1822G and p.E1834G). Using the Mutation Taster *in silico* prediction for c.5474C>T, the variant is assumed to be tolerated (data not shown).

### Coding mutation comparison of primary tumors and metastases

When analyzing the somatic mutational patterns of coding regions in each primary tumor and comparing it to the corresponding lymph node metastasis, several observations were made. Although an overlap was noted for all paired tumors and metastases suggesting a genetic linkage between the lesions, most mutations found in the metastases were not present in the primary lesion. Moreover, not all somatic mutations found in the primary tumor were transferred to the metastatic lesion. Of the 85 somatic protein-altering and splice-site SNVs found among the primary pPTCs, 65 (76%) were unique to the primary tumors, whereas 54 out of the 75 SNVs (72%) were exclusively found in the metastatic samples. Hence, the total shared mutational load between primary tumors and metastases was 20 SNVs, on average 4 per pair, compared to 13 and 10.8 SNVs on average unique to the primary lesion and metastasis, respectively (Fig. 1). These data suggest that the metastatic clone diverged early from the primary tumor, allowing additional mutations to develop independently in both lesions. A deeper analysis of clonality using whole-genome and copy number data was attempted using Sclust and PhyloWGS, but no reliable data was received given the exceedingly few genetic events in each tumor-metastasis pair.

### Gene ontology

To detect shared patterns among the mutated genes of importance in the tumor cohort, genes exhibiting somatic gene mutations were analyzed by the Reactome Pathway Database. The results are summarized in Table 2. While the most significantly represented processes varied among cases, two metastatic lesions (cases 4 and 8) both showed an overrepresentation of mutated genes associated to MCH class I antigen presentation.

**Table 2:** Gene ontology and expressional clustering of the five pediatric papillary thyroid carcinoma (pPTC) cases.

Case ID	1	2	3	4	5
Somatic driver event among established genes	None	*SQSTM1-NTRK3*	None	*BRAF* V600E	*NCOA4-RET*
Constitutional event*	*CHEK2* p.410fs	None	None	None	None
Mutational aggregation in primary tumor^#^	XBP1(S) activates chaperone genes	Translocation of ZAP-70 to immunological synapse	Activation of C3 and C5	RHO GTPase effectors	Depolymerization of the nuclear lamina
Mutational aggregation in lymph node met^#^	Glutathione conjugation	Antigen presentation (MHC I)	Stimuli-sensing channels	Frs2-mediated activation	Antigen presentation (MHC I)
RNA sequencing clustering of primary tumor	Cluster M	Cluster F	Cluster M	Cluster F	Cluster F
RNA sequencing clustering of metastasis	Cluster M	Cluster F	Cluster M	Cluster M	Cluster F

*Defined as a credible mutational event in a thyroid-related gene on the constitutional level; ^#^As highligthed by Reactome pathway analysis of mutated genes.

Cluster F, fusion cluster; cluster M, mutational cluster (both unsupervised clustering analyses).

### Germline variants

When analyzing sequencing data from constitutional DNA, case 1 was found to exhibit a heterozygote p.410 frameshift variant (rs555607708) in the DNA repair gene *Checkpoint kinase 2* (*CHEK2*) (Fig. 1). Constitutional mutations in this gene predispose to multiple tumors, including familial PTC (Cybulski *et al.* 2004, Siołek *et al.* 2015). According to the Catalogue of Somatic Mutations in Cancer (COSMIC), this specific variant (COSM5967258) has been previously predominantly reported in prostatic adenocarcinomas, and the minor allele frequency of this alteration in germline tissues is <0.2% (GnomAD), thereby arguing against a commonly observed SNP . For all other cases, no credible driver gene mutations in constitutional tissues were found.

### Gene fusion events

By filtering fusion events that were supported by analyses both on the DNA (Manta) and RNA (Kallisto fusion/Pizzly) levels, we obtained a list of credible fusion events that were expressed in each tumor sample (Fig. 1 and Supplementary Table 2). Two of the detected gene fusions have been previously reported in PTCs, *SQSTM1-NTRK3* (case 2, both primary tumor and metastasis) and *NCOA4-RET* (case 5, both primary tumor and metastasis) (Table 2). Case 4 displayed a *MIPOL1-SLC25A21* fusion in the primary tumor that was not carried over to the corresponding metastasis (Fig. 1). Additionally, case 2 also exhibited a *CEMIP-MTHFS* fusion in both the primary tumor and metastasis, a fusion that has not been previously associated with cancer. Moreover, a *CCDC32-CBX3* fusion was also noted in the primary tumors of cases 3, 4 and in the metastatic lesion of case 5. This fusion has been recently described in normal tissues, suggesting a passenger status (Singh *et al.* 2020) (Supplementary Table 2).

### Copy number alterations

The copy number alteration landscape of the pPTCs is detailed in Supplementary Fig. 6.

By analyses using the ASCAT software, both primary tumors and metastases were found entirely euploid without gross chromosomal alterations altogether.

### Non-coding events

Apart from the coding mutational panorama described above, we performed extended investigations of non-coding regions of potential interest for cancer development, by considering mutations in conserved regions with potential regulatory potential. No mutations in established promoter regions of clinical importance in PTC were seen (*TERT* promoter, *PLEKHS1*) (Alzahrani *et al.* 2016, Bournaud *et al.* 2019, Hysek *et al.* 2019, Xing *et al.* 2020). On average, 3.9 (range 0–14) somatic mutations affecting conserved bases in regulatory regions were detected (Supplementary Table 3). No regulatory region was mutated in more than one patient, and only four of the thirty-nine variants were found in both the primary and the metastatic lesions.

### RNA sequencing quality parameters

Ten samples (five primary pPTCs and five corresponding metastases) were successfully interrogated using RNAseq. The total million reads per sample were 73.86 on average, ranging from 52.99 to 89.14, and the average fragment size of the library was 394.15 bp, ranging from 358.03 to 432.15. The aggregated percentage of bases that had a quality score more than the Q30 value was 93.6% on average, ranging from 92.55% to 94.79%.

### Transcriptome analyses

**Figure 2:**
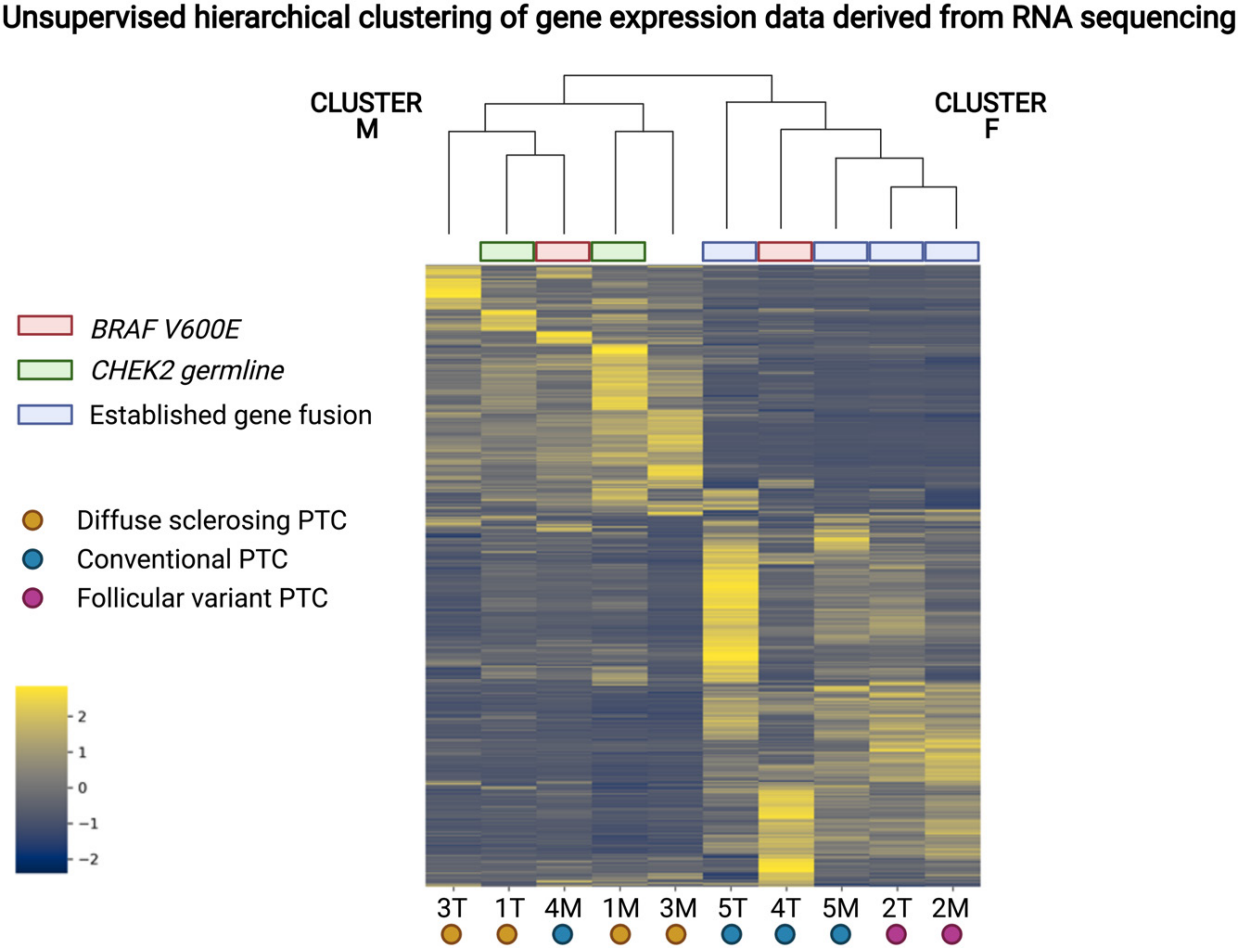
Unsupervised hierarchical clustering of gene expression data derived from RNA sequencing of individual pediatric papillary thyroid carcinomas (pPTCs). Two main clusters were identified; cluster M (mutation) and cluster F (fusion). In the cluster M, the sole *BRAF* mutant metastasis clusters with the tumors with a germline *CHEK2* mutation and the genetic 'orphan'. In cluster F, the fusion-related tumors cluster with the *BRAF* mutant primary tumor. Image created with BioRender.com.

Via RNAseq data, a gene expression heatmap was constructed using the 3000 most variable expressed genes within the cohort. By unsupervised clustering analyses, two main clusters were observed (Fig. 2 and Table 2). The fusion-driven primary and metastatic pPTCs (cases 2 and 5) clustered together (cluster F for 'fusion') with the metastatic *BRAF* mutated sample, while the primary *BRAF* mutated sample clustered together with the germline *CHEK2* mutated case and the sole genetic orphan (cluster M for 'mutation'). A differential expression analysis between both clusters was performed, and a significant overrepresentation of genes regulating various immunological processes was found (Table 3, Supplementary Fig. 7 and Supplementary Tables 4, 5). Moreover, when selectively analyzing the expression levels of 16 thyroid function and metabolism genes associated with thyroid cell differentiation – the so-called 'thyroid differentiation score' (Cancer Genome Atlas Research Network 2014) – a near-significant (*P* = 0.063) difference was found between clusters F and M, with a trend toward higher differentiation in cluster M (Supplementary Fig. 8). However, when comparing metastases and primary tumors, no genes were found differentially expressed.

**Table 3:** Top gene ontology (GO) terms enriched among the genes differentially expressed between clusters M and F.

GO term	Fold enrichment	FDR-corrected *P*-value
Negative regulation of antigen receptor-mediated signaling pathway (GO:0050858)	2.87	0.00832
Opsonization (GO:0008228)	2.86	0.00000196
Positive regulation of natural killer cell-mediated cytotoxicity (GO:0045954)	2.86	0.0368
Positive regulation of CD4-positive, alpha–beta T cell differentiation (GO:0043372)	2.8	0.0172
Negative regulation of lymphocyte apoptotic process (GO:0070229)	2.75	0.0144

**Figure 3:**
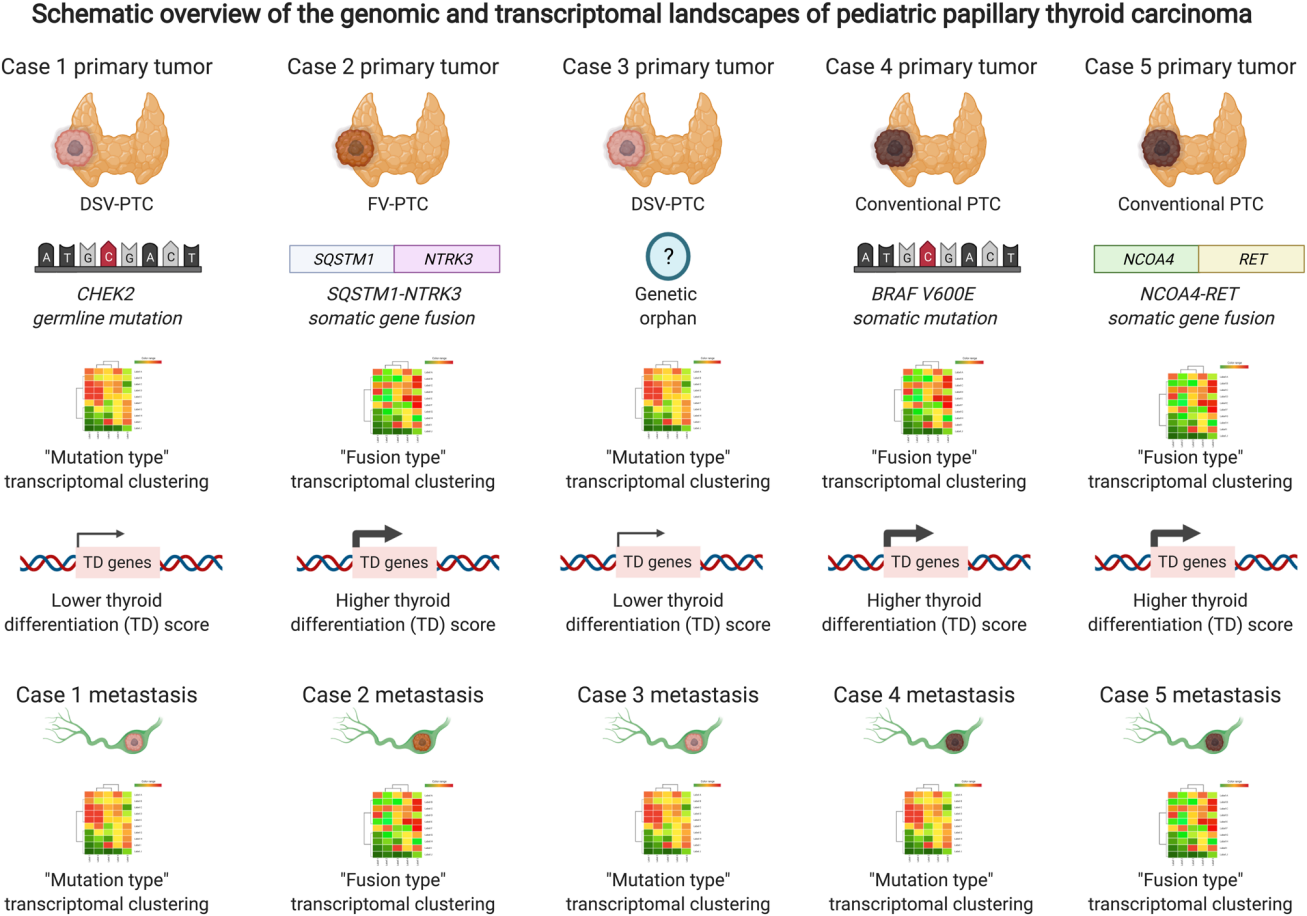
Schematic overview of the genomic and transcriptomal landscapes of pediatric papillary thyroid carcinoma. Cases are illustrated in terms of histological subtype, established driver gene events at either germline or somatic levels, transcriptome cluster adherence for both primary and metastatic tumors as well as the thyroid differentiation (TD) score. Image created with BioRender.com.

Our summarized findings from detailing the pan-genomic landscape of pPTC are schematically illustrated in Fig. 3.

### Extended *in silico* analyses in cases without credible somatic events

Two cases in our study, cases 1 and 3, lacked credible somatic genetic events coupled to established thyroid-related genes. Therefore, we performed extended *in silico* analyses in which all genes with somatic mutations in either the primary tumor or associated metastasis of these two cases were assessed in 414 independent PTC specimen from the cBioPortal for cancer genomics (TCGA) (Cancer Genome Atlas Research Network 2014). We evaluated each candidate gene from our study in terms of mutations, copy number alterations and mRNA expression in this large cohort, and also correlated any eventual findings to patient survival. In all, 388 PTCs were available for all analyses. By analyzing the TCGA mutational data, we observed missense mutations in the following genes overlapping with the genes mutated in case 1 of our study: *AKNA* (one TCGA cohort mutation; D1391Y), *FCGBP* (two TCGA cohort mutations; S201L and R4855C), *KDM6B* (two TCGA cohort mutations; S399T and A884V) and *OR11H12* (one TCGA cohort mutation; W164L). None of the mutated genes in case 3 were reported in the TCGA database, and neither of the mutated genes observed in case 1 or 3 were associated with patient survival when comparing each gene separately for copy number alterations or mRNA dysregulation in the TCGA cohort (data not shown).

### Case 1 gene candidates at the somatic level

**Figure 4:**
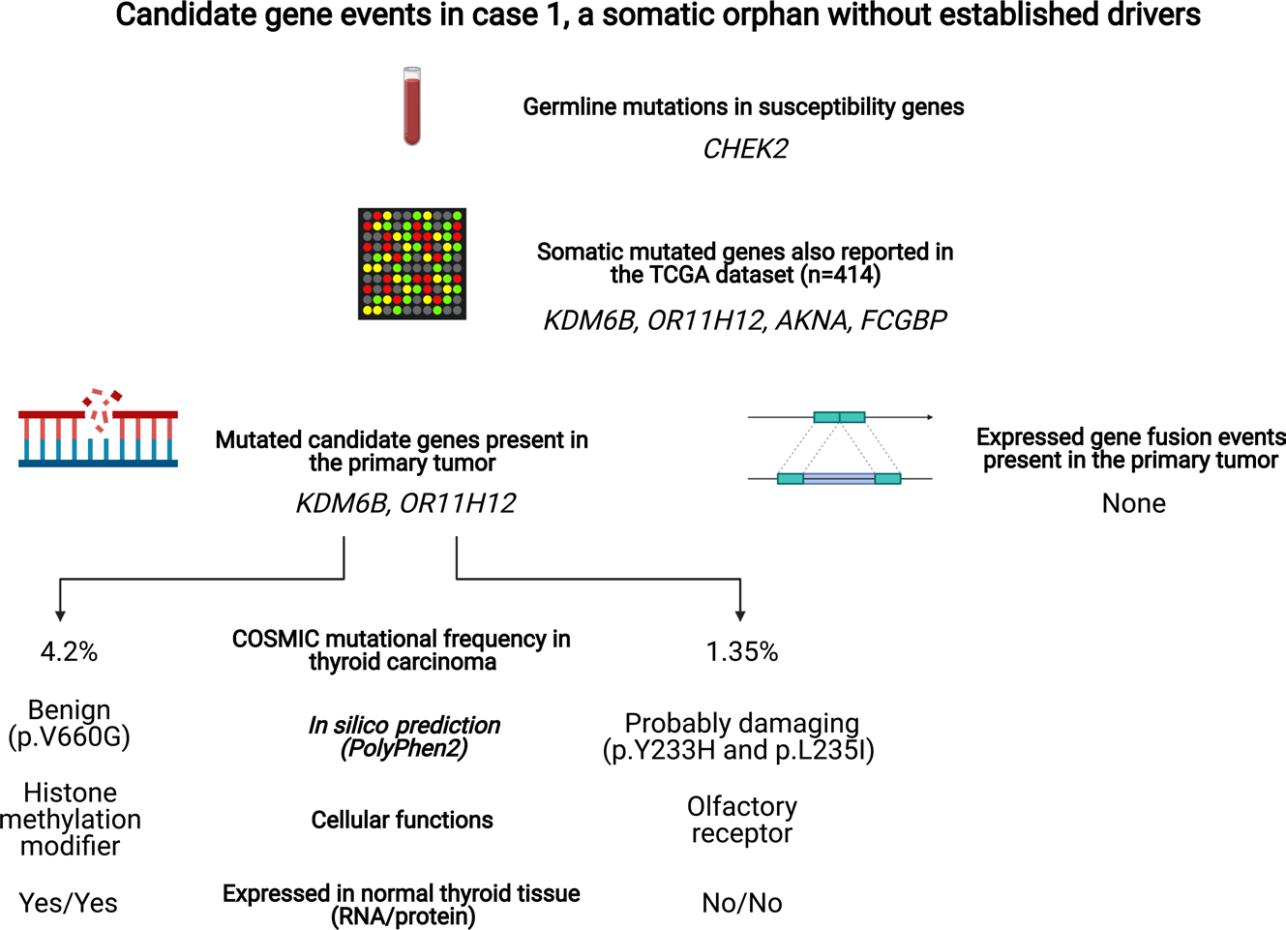
Candidate gene events in case 1, a somatic orphan without established drivers. Schematic illustration of genetic events of possible implications for tumor development in case 1. Although this case harbored a truncating constitutional *CHEK2* mutation, no credible somatic events were noted in thyroid-related genes. *KDM6B* and *OR11H12* were two mutated genes also found mutated in the TCGA database of PTCs. Image created with BioRender.com.

Case 1 exhibited a germline *CHEK2* mutation but no established somatic events. Since we looked for potential driver gene events of importance for the development of pediatric PTC, we mainly focused on alterations found in the primary tumor. Our results are summarized in Fig. 4. As the *AKNA* and *FCGBP* mutations in case 1 described above were only found in the metastatic tissue, we found these genes of limited importance for this particular topic (although not excluding them as relevant for tumor progression). In terms of events possibly influencing primary tumor development, *Lysine (K)-Specific Demethylase 6B* (*KDM6B)* caught our attention given its role as an epigenetic regulator and a histone methylation modifier. By demethylating di- or tri-methylated lysine 27 of histone H3 (H3K27me2 or H3K27me3), KDM6B controls chromatin organization and influences gene silencing. *KDM6B* is considered a tumor suppressor gene recurrently down-regulated in pancreatic adenocarcinoma (Yamamoto *et al.* 2014), while reported to exhibit oncogenic properties in ovarian cancer (Liang *et al.* 2019). The gene has also been found mutated in single cases of anaplastic thyroid carcinoma (Wagle *et al.* 2014). *KDM6B* expression is found in normal thyroid tissue, both on the RNA and protein level (https://www.proteinatlas.org/). In the COSMIC database, the *KDM6B* mutational frequency is 4.2% (44 mutated thyroid carcinomas out of 1040 tested, accessed Feb 7th, 2021). Our case (case 1) exhibited a somatic p.V660G missense mutation, which was not found among the 44 mutated cases in the COSMIC database and the mutation is predicted to be 'benign' as per PolyPhen2 *in silico* prediction (score 0.01). Thus, although reported as mutated in thyroid cancer, including PTCs from the TCGA database, we lack evidence that the sole *KDM6B* mutation found in one of our genetic orphans is indeed a driver of tumor development, which is also supported by the lack of this mutation in the corresponding lymph node metastasis.

We then turned to *OR11H12*, an olfactory receptor gene. The olfactory receptor gene family is the largest in the genome, and mutations in these genes are recurrently reported in human cancer. The *OR11H12* gene exhibited two somatic missense mutations in the primary tumor tissue of case 1 (p.Y233H and p.L235I) and was found mutated in a single PTC case from the TCGA dataset. Mutations in this gene have been reported in 1.35% (14 out of 1040) thyroid carcinomas according to the COSMIC database, accessed Feb 7th, 2021). The Y233H missense mutation was not reported in the dataset, but a truncating mutation at this codon was reported in a single PTC. Using PolyPhen2, this variant was classified as 'probably damaging' (score 1.0). Even more interestingly, the p. L235I mutation has been reported in five tumors (three PTCs and two unspecified 'thyroid carcinomas'), making it a recurrent genetic event in PTC. Among these five COSMIC mutated cases, the age at surgery was retrievable only for two cases; both these patients were 36-year-old females. The p. L235I mutation is denoted as 'probably damaging' via PolyPhen2 *in silico* analyses (score 0.989). However, as *OR11H12* is not known to be expressed in normal thyroid tissues when consulting the Human Protein Atlas, the functional consequences of these mutations are obscure.

In terms of non-coding mutations in regulatory regions, case 1 only displayed two somatic mutations, one in the primary tumor and a separate mutation in the corresponding metastasis, thereby arguing against an important role for these events. As no expressed fusion events were reported for case 1, we re-analyzed the fusion landscape for the complete set of chromosomal alterations, unfiltered in terms of RNA expression, and focused on Cancer Gene Census genes with potential implications for tumor development. Few events were noted in total, and although a few genes of interest were discovered as included in larger spans of genomic deletions, they were not reproduced by the ASCAT copy number analyses – possibly reflecting artefacts (data not shown).

### Case 3 gene candidates at the somatic level

**Figure 5:**
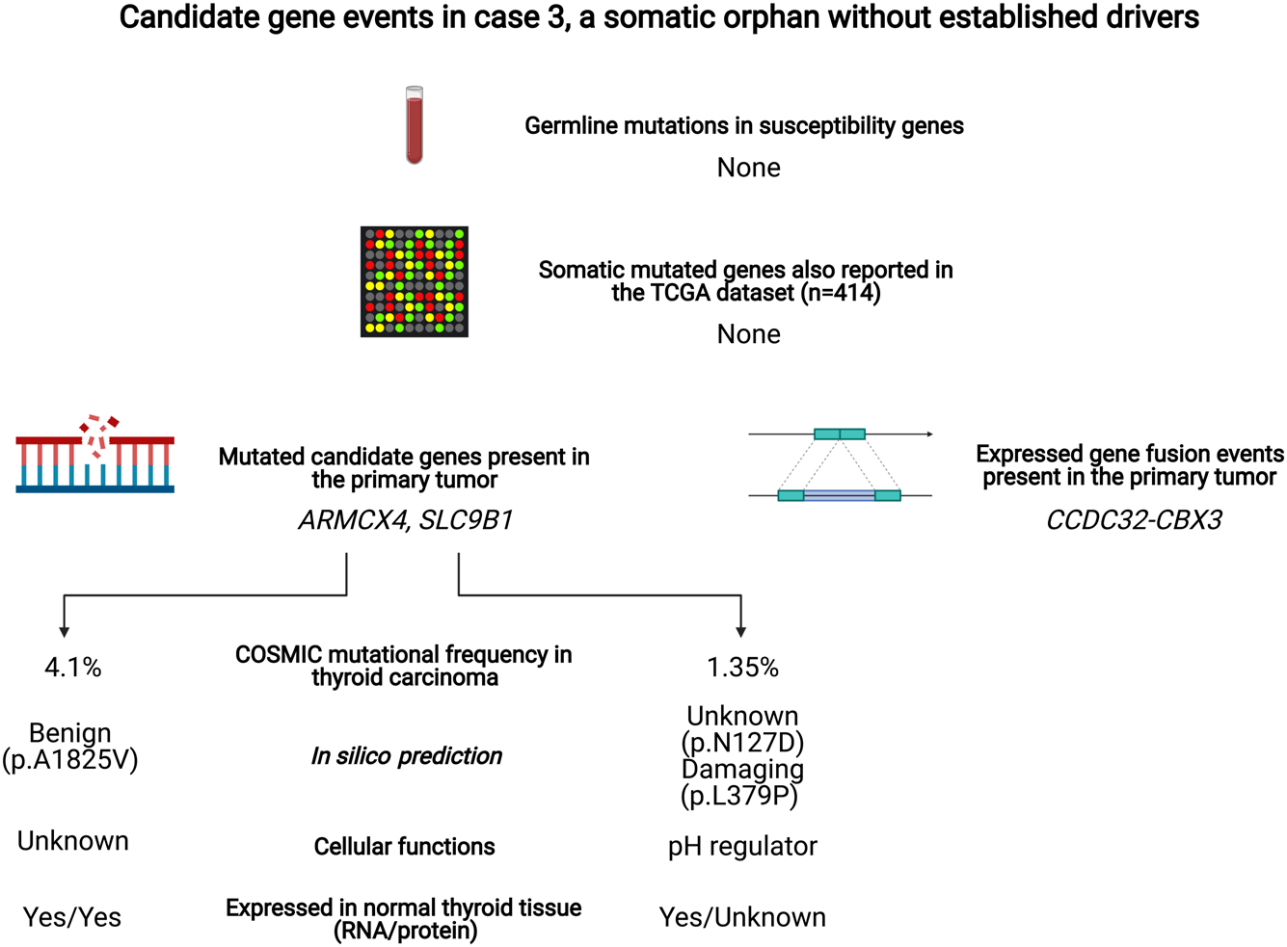
Candidate gene events in case 3, a somatic orphan without established drivers. Schematic illustration of genetic events of possible implications for tumor development in case 3. As no gene with somatic mutations in case 3 was reported as mutated in the TCGA database of PTCs, we manually scrutinized all possible candidate genes. Image created with BioRender.com.

This case exhibited no credible genetic events on either germline or somatic levels that could be coupled to any thyroid-related gene, and none of the somatic mutations in the primary or metastatic tissue were represented in the TCGA cohort of PTCs. After scrutinizing the individual mutations found in the primary tumor of this case, we here summarize potential candidate genes of interest. The findings are also illustrated in Fig. 5.

First, we choose to highlight a somatic p.Ala1825Val *ARMCX4* mutation. *ARMCX2* encodes a protein belonging to the armadillo repeat-containing family, with largely unknown functions. According to the Human Protein Atlas, the expression of this gene is abundant in the thyroid gland both on mRNA and protein levels. Although recurrently mutated in thyroid cancer specimens according to the COSMIC database, our specific somatic mutation has not been previously reported, and since the effect on protein function was expected to be low, we lack the evidence needed to designate this alteration as potentially causative of pPTC. Even so, this case clustered with the mutation-driven pPTCs, and no credible fusion gene event was noted either – findings which partly support the presence of a disease-causing mutation.

Moreover, we found two somatic mutations in the *SLC9B1* gene that were both present in the primary tumor as well as in the corresponding metastasis. *SLC9B1* encodes a membrane-bound sodium-hydrogen exchanger that regulates intracellular pH levels. According to the Human Protein Atlas (https://www.proteinatlas.org/), the gene is expressed in normal thyroid follicular epithelium as well as in thyroid cancer. According to the COSMIC database, 14 thyroid carcinomas with *SLC9B1* mutations are reported out of 1040 cases tested; 1.35%). Most of these mutations seem to be either truncating or missense alterations, vaguely arguing for a tumor suppressor function. Our case exhibited a p.Asn127Asp as well as a p.Leu379Pro missense mutation. While the former variant is of unknown significance, the latter is 'damaging' (PolyPhen2 score 1.0). By analyzing the individual reads for *SLC9B1*, both mutations were found in the same set of reads, thereby suggesting that they are present on the same allele (data not shown). This would argue against a biallelic type of mutational inactivation, which might diminish the biological significance of our findings. Moreover, the exact mechanism as to how a damaging mono-allelic mutation in a pH regulator would drive PTC tumorigenesis is unknown, although intracellular variations in pH levels might bear tumorigenic consequences in unrelated tumor types (Damaghi *et al.* 2013). There were no overt signs of *SLC9B1* expressional dysregulation that associated with any clinical parameters in the TCGA database of PTCs, thereby strongly limiting the value of additional wet lab expressional studies of this candidate.

In terms of gene fusions detected both by MANTA and RNA sequencing (expressed fusion genes), case 3 was found to harbor a *CCDC32-CBX3* fusion. The same fusion is reported in the primary tumor of case 4 and in the metastatic deposit of case 5. According to FusionHub, this phenomenon is reported also in various normal tissues and this, therefore, believed to constitute a non-pathogenic event. Indeed, the *CCDC32-CBX3* transcript is probably due to a polymorphic retrocopy of *CBX3* located in an intron of *CCDC32* (Cheetham *et al.* 2020). By analyzing all MANTA detected fusion calls (irrespectively of the status using RNA sequencing), a similar outcome as for case 1 was seen. Few events overall and larger deletions were not reflected by the ASCAT copy number analyses – suggestive of artefacts. In this study, we therefore chose to only present fusion events that were also supported by the RNA sequencing.

## Discussion

For many years, clinical approaches to children with thyroid cancer were based on experiences from treating adult patients with the same condition. However, as pediatric cases display unique attributes in terms of presentation, underlying genetic drivers and overall outcome, this patient category clearly demands specific guidelines (Francis *et al.* 2015, Lebbink *et al.* 2020). To better understand why certain patients with pPTC exhibit such an aggressive clinical course, we aimed to characterize a set of primary tumors and matched metastases using a pan-genomic approach. We found a near-placid genome overall, with exceedingly few coding mutational and gross chromosomal events, thus confirming previous observations of PTCs being genetically stable tumors.

We observed credible somatic driver gene events in three out of five cases, and additionally one case with a truncating, heterozygous germline variant in the *CHEK2* gene. Specifically, two fusion-driven pPTCs were detected, a *SQSTM1-NTRK3* fusion in case 2 and a *NCOA4-RET* fusion in case 5, while case 4 exhibited a *BRAF* V600E mutation. The somatic events have been thoroughly described in PTCs and were thus considered *bona fide* driver events in our cohort (Henke *et al.* 2014, Picarsic *et al.* 2016, Mostoufi-Moab *et al.* 2018, Pekova *et al.* 2020). The constitutional *CHEK2* variant described above in case 1 was also considered a possible culprit event, as germline mutations in this gene predispose for various cancers, including familial PTC (Cybulski *et al.* 2004, Siołek *et al.* 2015). Even so, no somatic alteration in any cancer-related gene was found in this sample, and we, therefore, speculate whether the *CHEK2* mutation found here would abrogate the ability of the CHEK2 protein to stabilize P53. Indeed, data from kindred with familial PTC exhibiting an unrelated truncating *CHEK2* mutation suggest that this event leads to diminished intratumoral P53 levels (Zhao *et al.* 2020). Even so, both case 1 as well as case 3 was without credible somatic driver alterations in PTC-related genes altogether. Although alterations in several candidate genes were highlighted, we lack the confidence to state that either one of these alterations was crucial to tumor development (Figs 4 and 5). This was not least solidified by the low abundance of similar alterations in the TCGA database.

The mutational panorama of primary tumors differed from their corresponding metastases, with few shared events, suggestive of an early divergence of the metastatic clone in each case. As two out of five primary tumors were multifocal, we acknowledge the risk that the metastatic lesion interrogated in this study might not be stemming from the sampled primary tumor for these cases – however, as these cases (3 and 5) shared two and five mutations respectively between the primary and metastatic lesions, we suspect that also these cases are genetically linked. Our findings mirror recent data in adult cases suggesting that primary PTCs and their corresponding metastasis only share subsets of mutations (Masoodi *et al.* 2020). Therefore, molecular analyses for medical considerations (either diagnostic or therapeutic) could in theory give rise to different results if the testing is targeting the metastatic PTC tissue rather than the primary lesion, which also should be clinically more relevant. Overall, since our cohort represents pPTCs with an aggressive clinical behavior, we also looked specifically for potential genetic aberrancies suitable for molecular targeted therapy. Indeed, the *SQSTM1*-*NTRK3* fusion in case 3, the *BRAF* V600E mutation in case 4, and the *NCOA4*-*RET* fusion in case 5 are all druggable targets using TRK, BRAF and tyrosine kinase/RET inhibitors respectively, and all three events were observed also in the metastatic tissues. As our WGS analysis did pinpoint important therapeutic targets in the majority of clinically advanced pPTCs, these findings highlight the potential value of multi-gene sequencing also for pediatric thyroid cancer cases.

**Figure 6:**
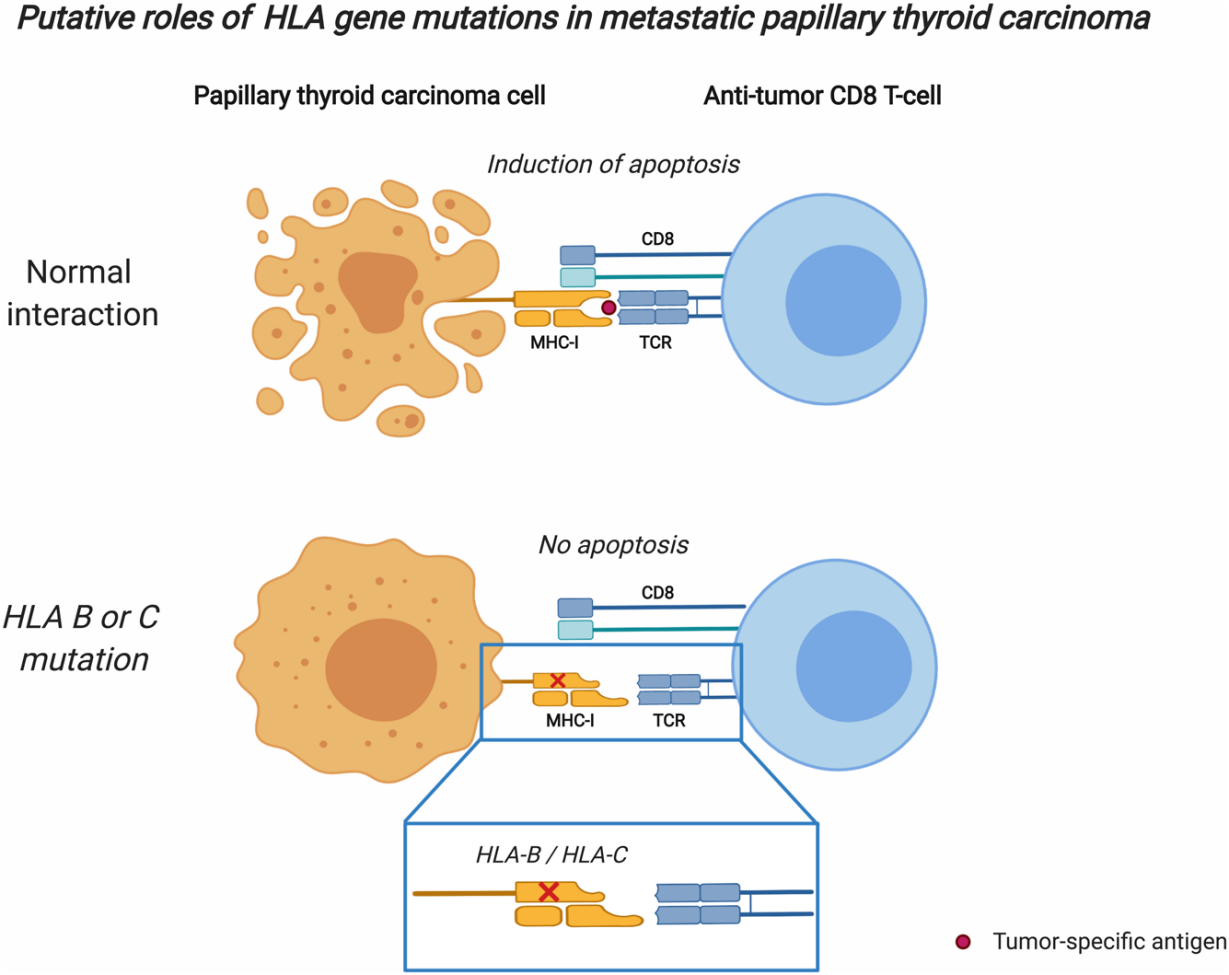
Putative roles of HLA gene mutations in metastatic papillary thyroid carcinoma. This is a schematic overview of the natural crosstalk between papillary thyroid carcinoma (PTC) and a CD8+ T cell, depicting a PTC cell exhibiting a tumor-specific antigen via MHC class I receptors (encoded by *HLA* genes), leading to an activation of the T cell, thereby inducing apoptosis of the cancer cell. In the bottom part, *HLA* gene mutated cells are shown. As they theoretically exhibit an impaired MHC class I interaction with T cells, this could be a way to avoid the induction of apoptosis. TCR; T cell receptor. Image created with BioRender.com.

From a gene ontology perspective, we observed an overrepresentation of mutations in genes associated with MHC class I antigen presentation in two out of five metastatic samples. Interestingly, both these cases were fusion-driven pPTCs (cases 2 and 5), of which one later recurred with distant metastases. Of note, fusion-driven pPTCs clustered together using expressional data from the RNAseq, and this expressional pattern largely involved genes involved in immune system regulation. Indeed, lower expression of immune response genes has previously been reported in PTCs (Cancer Genome Atlas Research Network 2014). Thus, it is tempting to speculate that the metastatic potential of pPTCs with established fusion events in part is influenced by secondary mutations in genes responsible for communication with immune cells, not least evident by the finding of somatic mutations in *HLA-B* and *HLA-DRB1* (case 2, metastatic lesion) and *HLA-C* (case 5, metastatic lesion). *HLA-B* and *HLA-C* are two of the major MHC class I genes, encoding proteins that display peptide fragments to circulating immune cells. Hypothetically, mutations in these genes could potentially interfere with the communication between tumor cells and the immune system (Fig. 6). Interestingly, functional studies have shown that augmented MCH class I expression in PTC cell lines alters the immune response and incapacitates the ability of immune escape (Hu *et al.* 2020, Lu *et al.* 2020). As our theory builds on two cases only, more research is needed to elucidate if the *HLA* mutations detected in our cohort indeed are clinically relevant.

The expressional profiles of pPTCs were dependent on the underlying genetic driver event, as evident by different clustering depending on whether the tumor was driven by gene fusion or mutation. Moreover, the fusion-driven tumors, in general, displayed a lower thyroid differentiation score (TDS) than mutation-driven samples, suggesting that gene fusion-positive pPTCs are less differentiated. This is vaguely mirrored by the clinical characteristics of the cohort, as the sole patient exhibiting distant metastases exhibited a fusion-driven tumor. Interestingly, the TCGA network did reveal lower TDS for *BRAF-*driven PTCs compared to *RAS* driven PTCs, and the former group was enriched for fusion-driven tumors as compared to the latter (Cancer Genome Atlas Research Network 2014). For case 4, the only *BRAF* driven tumor, discordant findings between primary and metastatic lesions were obtained – as the primary tumor clustered with the fusion-positive primaries and metastases, whereas the metastatic sample clustered with the other mutation-driven sample and the sole genetic orphan. The reason for this discrepancy is not known, but since the primary tumor and corresponding metastasis carry 18 and 19 unique (private) somatic mutations respectively, there is always a possibility that one or several of these genes influence the gene expression output. Moreover, we did identify a *MIPOL1-SLC25A21* fusion in the primary tumor of case 4 which was not found in the corresponding metastasis (Fig. 1). This fusion gene has previously been observed in TCGA analyses of lung adenocarcinoma and urinary bladder urothelial carcinomas, but to our knowledge never reported in thyroid cancer. Interestingly, *MIPOL1* is reported to exhibit tumor suppressor gene properties in nasopharyngeal carcinoma (Cheung *et al.* 2009). Future studies of this fusion will hopefully elucidate how frequently it occurs in pediatric thyroid cancer, and if it could influence the global expressional output in PTCs in a similar manner as *bona fide* fusions involving *NTRK* or *RET*. However, it should be stated that our clustering analysis is based on a small sample series, and therefore validations in larger clinical series is warranted.

We conclude that pPTCs are genetically indolent tumors with an overall stable genome. The divergent mutational panorama in primary tumors compared to metastases suggests an early divergence in the phylogenetic tree and might indicate that molecular testing of local metastases is more clinically relevant for therapeutic reasons than to analyze the primary tumor itself. Moreover, we identify an overrepresentation of mutations in tumor-immune crosstalk pathways among fusion-driven pPTCs that might highlight molecular mechanisms of interest that govern metastatic spread. Also, small subgroups of pPTCs are still considered genetic orphans at the somatic level even after pan-genomic analyses, possibly indicating that unknown epigenetic mechanisms or low-frequency mutations in unestablished genes influence the development of PTCs in children.

## Supplementary Material

Supplementary Figure1.

Supplementary Figure2.

Supplementary Figure3.

Supplementary Figure4.

Supplementary Figure5.

Supplementary Figure6.

Supplementary Figure7.

Supplementary Figure8.

Supplementary Table 1. Detailed information of all coding somatic variants in the pediatric PTC cohort

Supplementary Table 2. List of all gene fusion events in the pPTC cohort as called by MANTA and RNAseq

Supplementary Table 3. List of mutated regulatory regions in the non-coding part of the genome

Supplementary Table 4: Genes differentially expressed between clusters M and F

Supplementary Table 5: GO enrichment analyses of genes differentially expressed between clusters M and F

## Declaration of interest

The authors declare that there is no conflict of interest that could be perceived as prejudicing the impartiality of the research reported.

## Funding

The authors are grateful for the grant support from the Swedish Cancer Society, the Swedish Society for Medical Research and Karolinska Institutet.

## Author contribution statement

A S, J Z, and C C J conceived and designed the study as well as provided clinical details. A S, K J, J P, S B and C C J performed the experiments and carried out data analysis. S B performed the bioinformatics. C C J drafted the manuscript. All authors were involved in analyzing the data, writing and editing the paper and had final approval of the submitted and published versions.
