# Supplementary material for: Pan-genomic characterization of high-risk pediatric papillary thyroid carcinoma
Source: Endocr Relat Cancer. 2021 Apr 6;28(5):337–51. doi: 10.1530/ERC-20-0464 (PMC8111328; doi:10.1530/ERC-20-0464)
Supplement: Supplementary Figure1. [file supplementary_figure_1.pdf]

Supplementary Figure 1. Histopathological attributes of case 1 (diffuse sclerosing variant PTC).

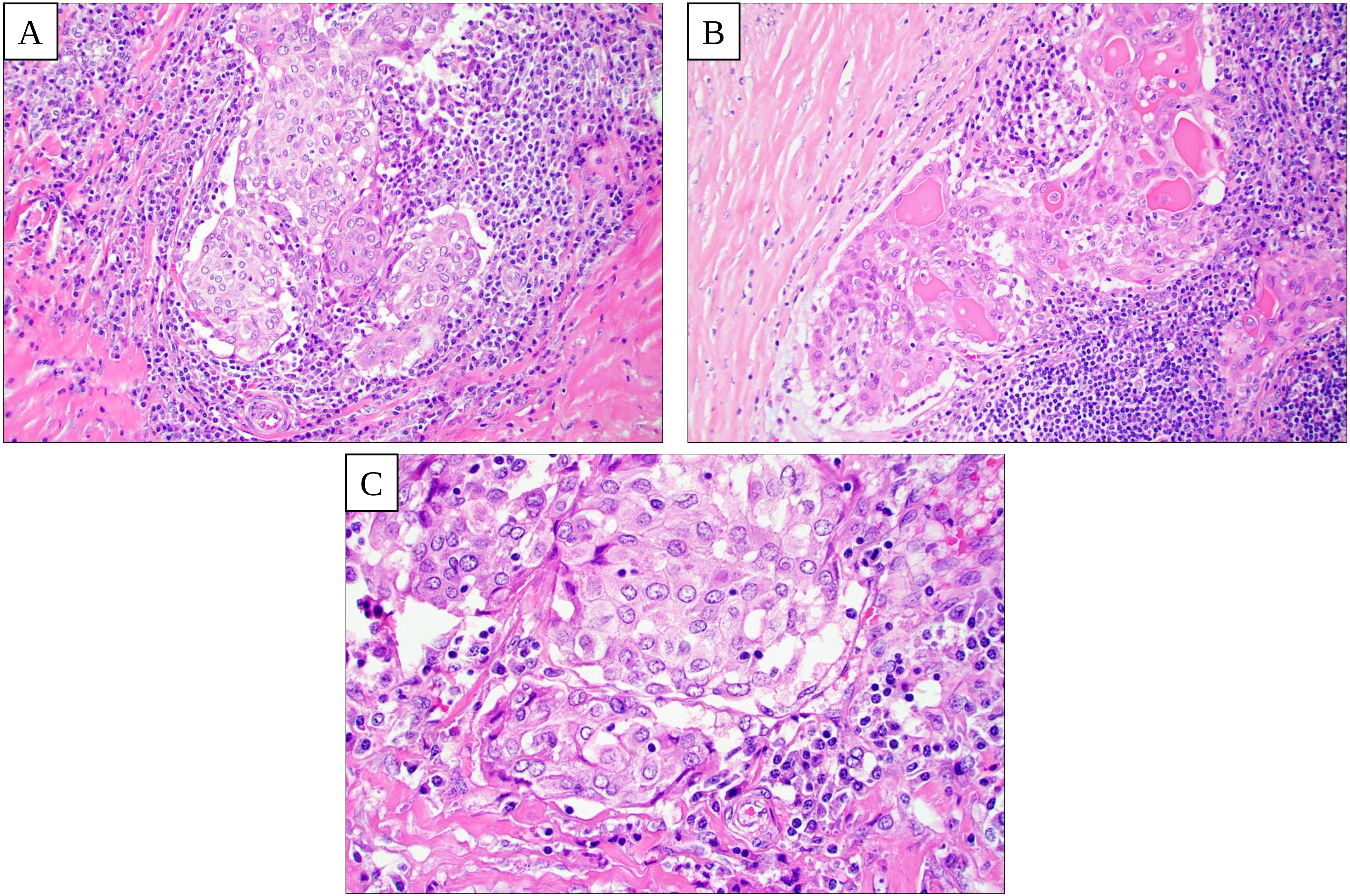

Hematoxylin-eosin stain at x200 magnification of the primary tumor (**A**) and corresponding lymph node metastasis (**B**), with an additional x400 magnification of the primary tumor (**C**). Note the associated inflammation, the focal squamous metaplasia and the adjacent fibrosis. This lesion also exhibited significant amounts of calcifications (not shown). PTC-associated nuclear changes are evident in image C.
