# Supplementary material for: Pan-genomic characterization of high-risk pediatric papillary thyroid carcinoma
Source: Endocr Relat Cancer. 2021 Apr 6;28(5):337–51. doi: 10.1530/ERC-20-0464 (PMC8111328; doi:10.1530/ERC-20-0464)
Supplement: Supplementary Figure5. [file supplementary_figure_5.pdf]

Supplementary Figure 5. Histopathological attributes of case 5 (conventional PTC).

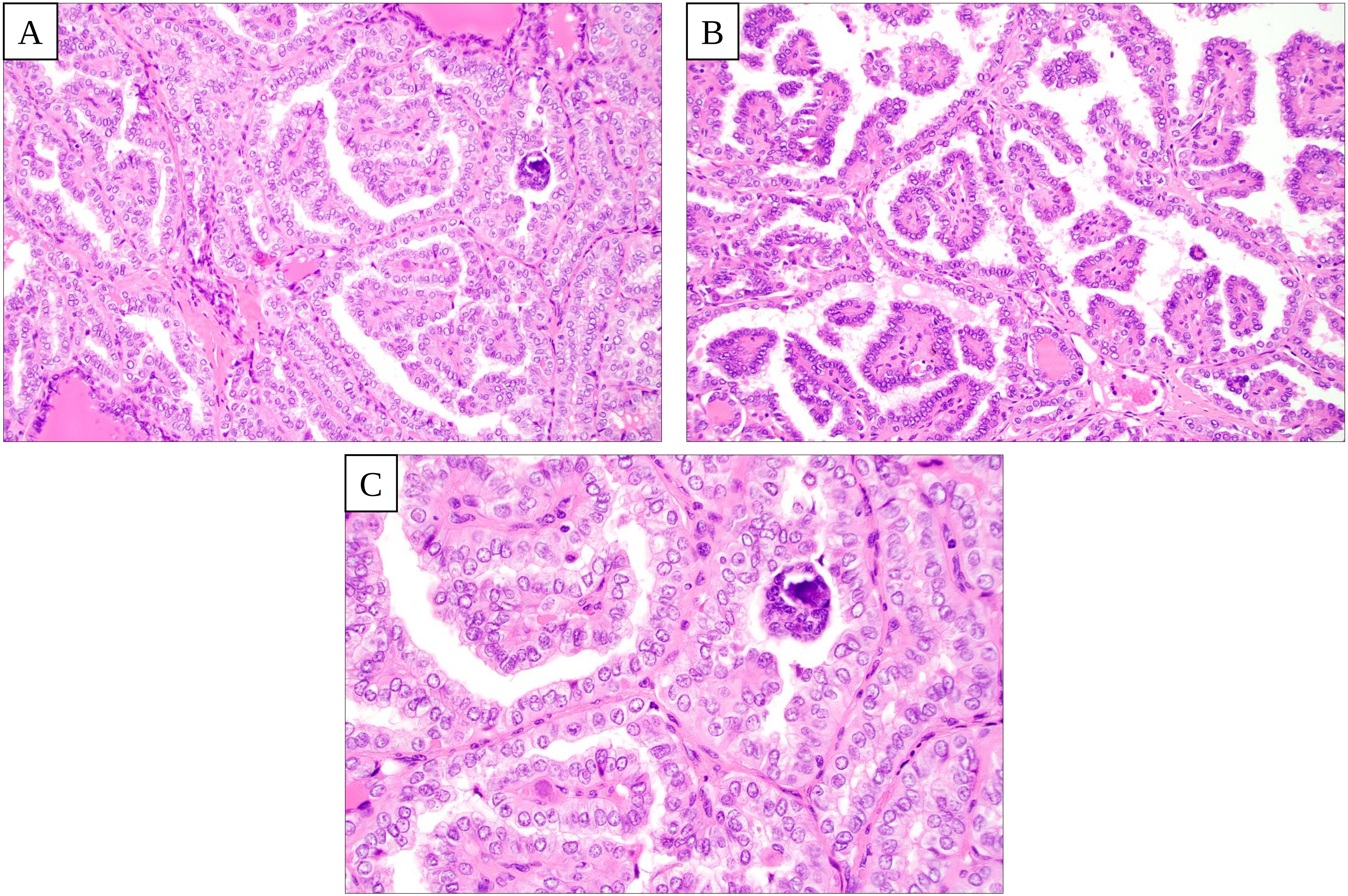

Hematoxylin-eosin stain at x200 magnification of the primary tumor (**A**) and corresponding lymph node metastasis (**B**), with an additional x400 magnification of the primary tumor (**C**). Conventional papillary growth with occasional psammoma bodies were observed. PTC-associated nuclear changes are evident in image C.
