# Supplementary material for: Pan-genomic characterization of high-risk pediatric papillary thyroid carcinoma
Source: Endocr Relat Cancer. 2021 Apr 6;28(5):337–51. doi: 10.1530/ERC-20-0464 (PMC8111328; doi:10.1530/ERC-20-0464)

Supplementary Figure 6 – ASCAT profiles for the 10 tumor samples, all predicted non-aberrant.

1T

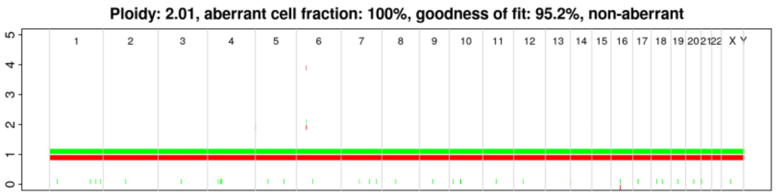

3T

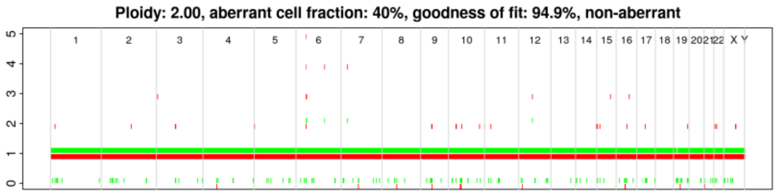

5T

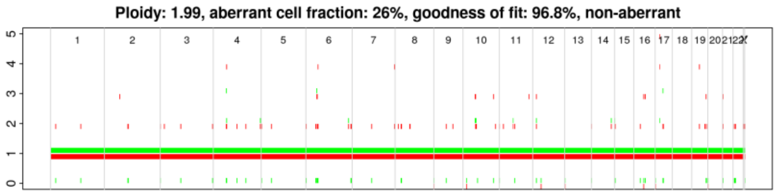

1M

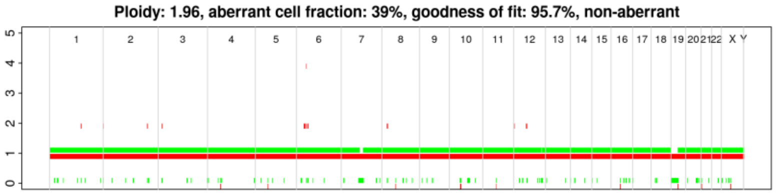

3M

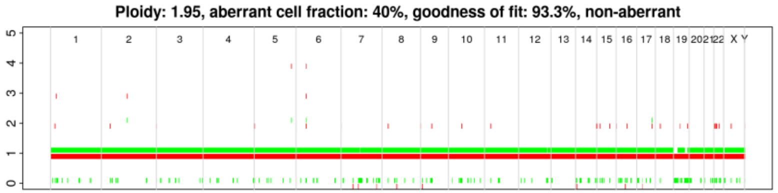

5M

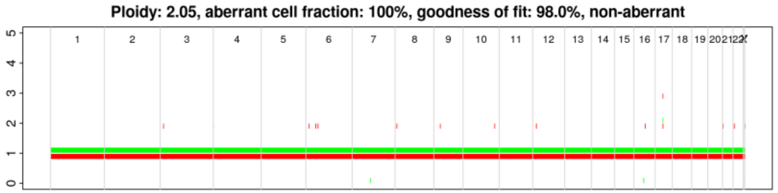

2T

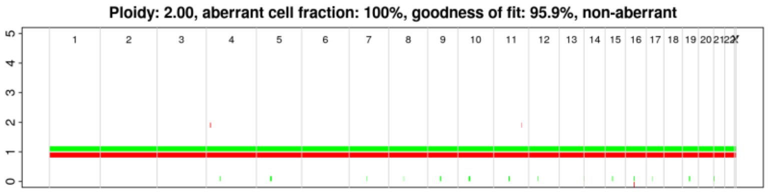

4T

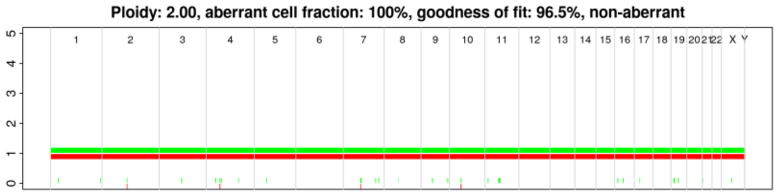

2M

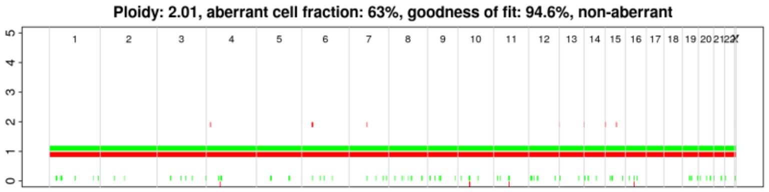

4M

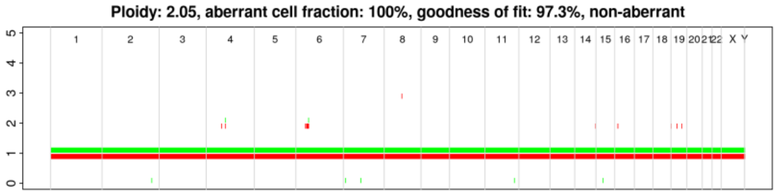

Supplement: Supplementary Figure6. [file supplementary_figure_6.pdf]
