# Supplementary material for: Pan-genomic characterization of high-risk pediatric papillary thyroid carcinoma
Source: Endocr Relat Cancer. 2021 Apr 6;28(5):337–51. doi: 10.1530/ERC-20-0464 (PMC8111328; doi:10.1530/ERC-20-0464)

Supplementary Figure 7. Volcano plot of the differential expression analysis between the two clusters M and F. 5786 differentially expressed genes (red) are detected. Genes with negative beta-value have higher expression in cluster F while genes with positive beta-value have higher expression in cluster M.

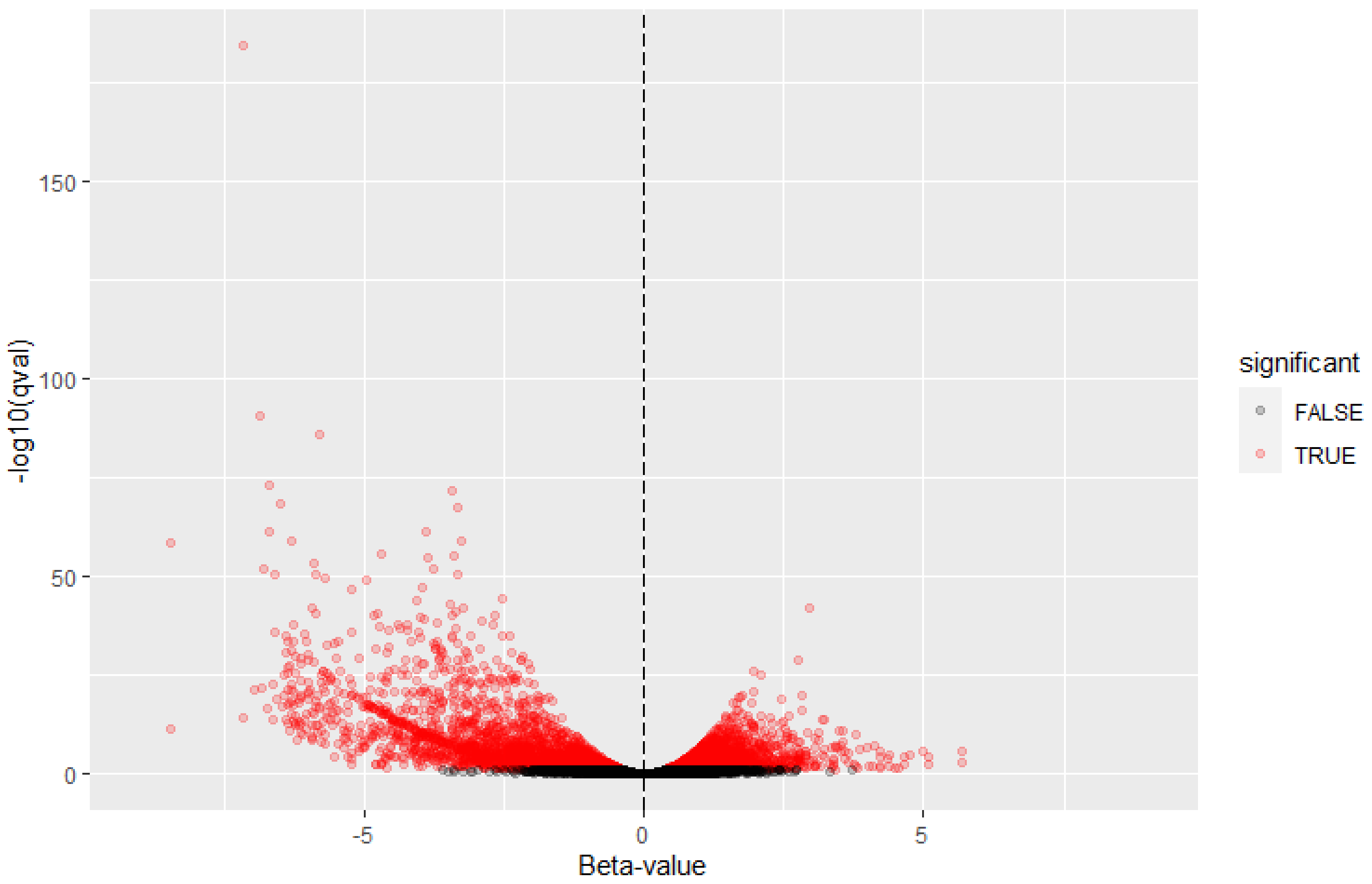

Supplement: Supplementary Figure7. [file supplementary_figure_7.pdf]
