# Supplementary material for: Pan-genomic characterization of high-risk pediatric papillary thyroid carcinoma
Source: Endocr Relat Cancer. 2021 Apr 6;28(5):337–51. doi: 10.1530/ERC-20-0464 (PMC8111328; doi:10.1530/ERC-20-0464)

Supplementary Figure 8. Thyroid differentiation score (TDS) in the two RNA expressional clusters.
TDS was calculated via expression levels of 16 thyroid metabolism and function genes, as defined by the TCGA consortium.
.

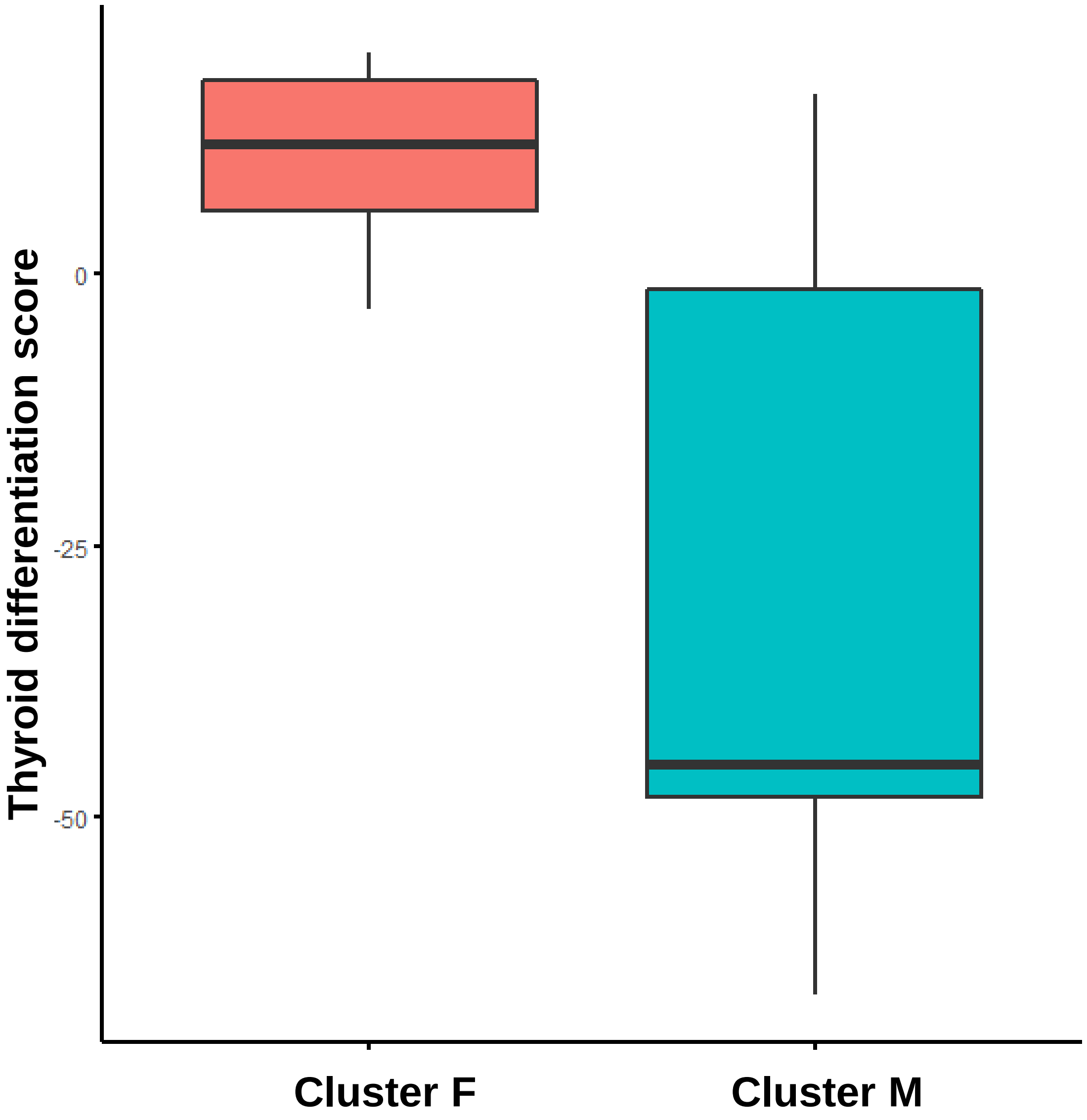

Supplement: Supplementary Figure8. [file supplementary_figure_8.pdf]
